# Supplementary material for: Observation of hybrid Tamm-plasmon exciton- polaritons with GaAs quantum wells and a MoSe2 monolayer
Source: Nat Commun. 2017 Aug 15;8:259. doi: 10.1038/s41467-017-00155-w (PMC5557755; doi:10.1038/s41467-017-00155-w)
Supplement: Supplementary file 1 — Supplementary Figures, Supplementary Notes and Supplementary References [file 41467_2017_155_MOESM1_ESM.pdf]

File name: Supplementary Information
Description: Supplementary Figures, Supplementary Notes and Supplementary References

File name: Peer Review File
Description:

Supplementary Note 1: Transition energies

We present simulations of electron – heavy hole transition energies in GaAs/AlAs - QWs at the gamma point and photoluminescence of a $MoSe_2$ - monolayer on a GaInP – surface (Supplementary Fig 1). The photoluminescence peaks of the monolayer correspond to its exciton and trion – energies. The measurement was taken on a sample comparable to the hybrid polariton base structure without embedded QWs. The simulations in Fig. verifies, that we need 5 nm thick GaAs/AlAs QWs with a high precession for a device which is both resonant to the $MoSe_2$ trions and excitons.

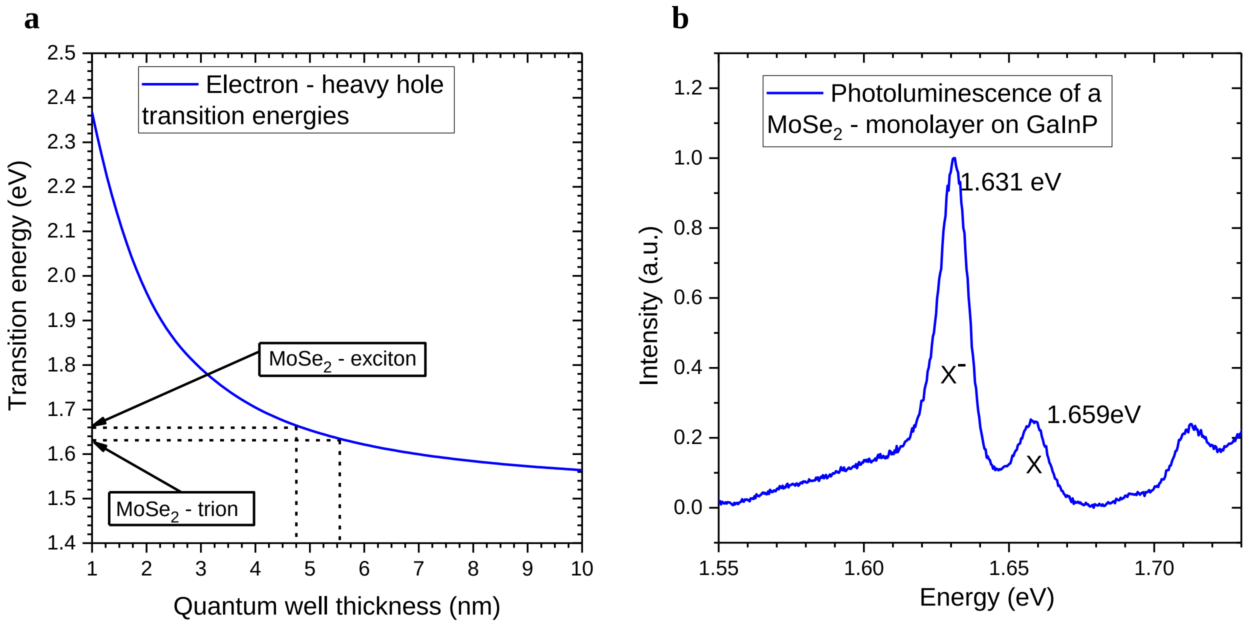

Supplementary Figure 1|: Transition energies of GaAs QWs and $MoSe_2$ excitons

a, Calculated electron – heavy hole transition energies at the gamma point of GaAs/AlAs – QWs.

b, Photoluminescence measurement of a $MoSe_2$ – monolayer on a GaInP – surface.

Supplementary Note 2: Photonic fraction of middle polariton branch

Here we investigate the temperature dependency of the photonic Hopfield coefficient of the middle polariton branch in our device. The temperature dependent absorption of the $MoSe_2$ – monolayer and the GaAs – QWs are shown in Fig S2a. The Varshni fit parameters are in good agreement with the literature [1,2] ($\alpha_{MoSe_2} = 4.12 * 10^{-4}$eV/K, $\beta_{MoSe_2} = 137.7$ K, $\alpha_{GaAs_2} = 5.405 * 10^{-4}$ eV/K,

$\beta_{GaAs} = 204.0$ K).

Supplementary Fig 2 depicts the temperature dependent detuning of the $MoSe_2$ and GaAs – excitons in our sample, which yields an increasing photonic Hopfield coefficient of the middle polariton branch in k=0. We observe, that the Hopfield coefficient is very small below 120 K and increases strongly after 150 K, which also explains, that this branch is dark at our 4 K and 140 K angle resolved PL measurements.

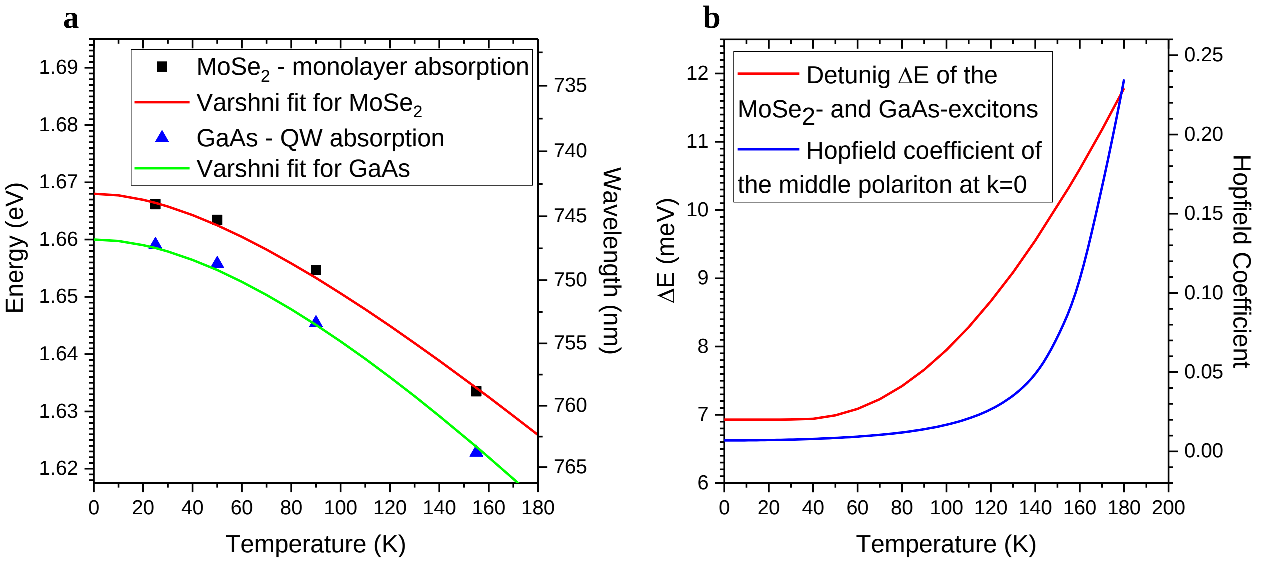

Supplementary Figure 2|: Photonic fraction of middle polariton branch

a, Temperature dependent absorption of the GaAs – QWs and $MoSe_2$ – monolayer. **b,** Temperature dependent detuning of the $MoSe_2$ – and GaAs – excitons and temperature dependent behavior of the photonic Hopfield coefficient of the middle polariton branch in k=0.

References

1. Lundt, N. *et al.*, Monolayered MoSe2: a candidate for room temperature polaritonics. *2D - materials* **4,** 1 (2016)

2. Vurgaftman., I., Meyer, J. R., Ram-Mohan, L.R. Band parameters for III-V compound semiconductors and their alloys. *Journal of Applies Physics* **89,** 5815 (2001).
