# Supplementary material for: Observation of hybrid Tamm-plasmon exciton- polaritons with GaAs quantum wells and a MoSe2 monolayer
Source: Nat Commun. 2017 Aug 15;8:259. doi: 10.1038/s41467-017-00155-w (PMC5557755; doi:10.1038/s41467-017-00155-w)
Supplement: Supplementary file 2 — Reviewer reports and authors' response from the peer review of this Article at Nature Communications [file 41467_2017_155_MOESM2_ESM.pdf]

Reviewers' Comments:

Reviewer #1:
Remarks to the Author:
The manuscript "Observation of hybrid polaritons in the regime of collective strong coupling of Tamm plasmons with excitons in GaAs quantum wells and MoSe2 monolayers" by M. Wurdack, N. Lundt, M. Klaas, V. Baumann, A. Kavokin, S. Höfling, and C.Schneider1 is devoted to the studies of the formation of hybrid exciton-polaritons in the collective regime of strong coupling between Wannier type of excitons in GaAs quantum wells, strongly bound valley excitons in a MoSe2 monolayer and cavity photons in a Tamm-plasmon-polariton device. The three characteristic hybrid polariton resonances were observed, and their occupation was explained by a thermodynamic model. In the manuscript, the observation of this new kind of quasi-particles is reported, and the manuscript provides some suggestions for applications of hybrid exciton-polaritons. According to the manuscript, while the monolayer excitons are robust and allow to maintain the spinor degree of freedom, the GaAs Wannier QW excitons are sensitive to external magnetic and electric fields, and support lasing and condensation effects in the strong coupling regime. The energy and k-vector distribution of exciton-polaritons along the hybrid modes by a thermodynamic model, The authors claim that a straightforward development of the presented device would enable direct electrical injection into the hybrid polariton states in a standard LED configuration.

In summary, the results of the studies presented in this manuscript are new, exciting, and timely. The theoretical approach used for calculations corresponds to the modern level of the theory. I recommend this manuscript for publication in Nature Communications.

Reviewer #2:
Remarks to the Author:
The paper presents a study of exciton-polaritons in a system composed by multiple GaAs/AlAs quantum wells (QWs) with a monolayer MoSe2 on top. Although the topic is not really new separately for each case, the combination of these two parts (namely, a III-V semiconductor heterostructure and a monolayer TMDC) is somewhat new and brings interesting results and novel possibilities for tuning light-matter interactions through hybrid exciton-polaritons.

Experimental results are clear and the theoretical model matches very well with them. Part of the features in the experimental observations are known to the literature and can be used to validate their results: (i) Exciton and trion peak positions for MoSe2 extracted from the results in Fig. 1d of the present paper are in good agreement with other previous experimental papers in the literature [see e.g. Nature Materials 16, 182 (2017)]; (ii) the coupling strength for MoSe2 exciton-polariton as obtained by a Rabi-like oscillator model is quite strong, of the order of 20 meV, which is in perfect agreement e.g. with Ref. [20]. In addition to those features, the observation of hybrid exciton-polaritons from the adjacent QWs coupled to the TMDC, as manifested by 3 modes in the PL spectra, well described by a 3 oscillators model, is an interesting and original result.

I therefore recommend the paper for publication in Nature Communications, but I still have some minor corrections to suggest:

- I understand References 20-22 are supposed to be for exciton-polaritons in TMDC monolayers, as stated in the introduction where these references are called, but Ref. [20] is more about van der Waals heterostructures. But most importantly, there is an earlier paper on this topic missing: Nature Photonics 9, 30 (2015), which was published (on-line) about a year before Ref. [20]. It is worth to cite it along with 20-22

- It would be better to define the abbreviation DBR before its use.

- Equations are not numbered. The first equation in the manuscript is a matrix equation. Then, just after it, there is a determinant equation: is this equation correct? shouldn't it have "-E" added to the diagonal terms? Anyway, in may opinion, showing this step on the diagonalization of the matrix in first equation is unnecessary - it's standard procedure.

Reviewer #3:
Remarks to the Author:
The authors demonstrated the strong light-matter coupling between the Tamm plasmons and the excitons in GaAs quantum wells and MoSe2 monolayers which lead to the formation of hybrid polariton modes. The momentum resolved photoluminescence and reflectivity results clearly show the anticrossing feature of such hybrid system. This may be useful for the design of the electrical injection of hybrid polariton LED device or polariton laser. Before it is publishable, I have some minor comments about it shown below.

1. In Fig. 2b, is the calculated reflectivity spectrum stands for the empty cavity mode? The reflectance dip of it corresponds to 1.64 eV is different from the black dashed line in Fig 3a at k//=0 (about 1.633 eV), where is the redshift comes from?
2. minor mistake: the label of the last picture in Fig. 4 is "b" which should be "f".
3. In Fig 3 and Fig. 4, the authors compared two different temperatures 4 K/150 K and 4 K/140 K, it would be better if the high temperature is the same.
4. Page 6, the meaning of the coefficient should be explained.
5. Minor mistakes in Supplementary materials:
First paragraph: "(Fig. S2b)" should be "(Fig. S1b)";
Third paragraph: "Fig. 2Sb" should be "Fig. S2b".

Reviewer #4:
Remarks to the Author:
The manuscript is an experimental demonstration of hybridization between QW excitons and 2D excitons in MoSe2 mono-layers placed in the cavity. The work is highly relevant and well performed. I recommend publication provided the authors answer a few comments
(1) How does the fact that the excitons in MQW's have longer lifetime (narrow line-width) influence hybridization?
(2) Will applying bias across the structure change hybridization as excitons go in and out of the resonance?
The authors claim that the hybrid polaritons have advantages when it comes to the electrically pumped LED, but this advantage is not clear to me.

Reviewer #1 (Remarks to the Author):

The manuscript "Observation of hybrid polaritons in the regime of collective strong coupling of Tamm plasmons with excitons in GaAs quantum wells and MoSe2 monolayers” by M. Wurdack, N. Lundt, M. Klaas, V. Baumann, A. Kavokin, S. Höfling, and C.Schneider is devoted to the studies of the formation of hybrid exciton-polaritons in the collective regime of strong coupling between Wannier type of excitons in GaAs quantum wells, strongly bound valley excitons in a MoSe2 monolayer and cavity photons in a Tamm-plasmon-polariton device. The three characteristic hybrid polariton resonances were observed, and their occupation was explained by a thermodynamic model. In the manuscript, the observation of this new kind of quasi-particles is reported, and the manuscript provides some suggestions for applications of hybrid exciton-polaritons. According to the manuscript, while the monolayer excitons are robust and allow to maintain the spinor degree of freedom, the GaAs Wannier QW excitons are sensitive to external magnetic and electric fields, and support lasing and condensation effects in the strong coupling regime. The energy and k-vector distribution of exciton-polaritons along the hybrid modes by a thermodynamic model, The authors claim that a straightforward development of the presented device would enable direct electrical injection into the hybrid polariton states in a standard LED configuration.

In summary, the results of the studies presented in this manuscript are new, exciting, and timely. The theoretical approach used for calculations corresponds to the modern level of the theory. I recommend this manuscript for publication in Nature Communications.

We thank the referee for his positive evaluation of the manuscript.

Reviewer #2 (Remarks to the Author):

The paper presents a study of exciton-polaritons in a system composed by multiple GaAs/AlAs quantum wells (QWs) with a monolayer $MoSe_2$ on top. Although the topic is not really new separately for each case, the combination of these two parts (namely, a III-V semiconductor heterostructure and a monolayer TMDC) is somewhat new and brings interesting results and novel possibilities for tuning light-matter interactions through hybrid exciton-polaritons.

Experimental results are clear and the theoretical model matches very well with them. Part of the features in the experimental observations are known to the literature and can be used to validate their results: (i) Exciton and trion peak positions for $MoSe_2$ extracted from the results in Fig. 1d of the present paper are in good agreement with other previous experimental papers in the literature [see e.g. Nature Materials 16, 182 (2017)]; (ii) the coupling strength for $MoSe_2$ exciton-polariton as obtained by a Rabi-like oscillator model is quite strong, of the order of 20 meV, which is in perfect agreement e.g. with Ref. [20]. In addition to those features, the observation of hybrid exciton-polaritons from the adjacent QWs coupled to the TMDC, as manifested by 3 modes in the PL spectra, well described by a 3 oscillators model, is an interesting and original result.

I therefore recommend the paper for publication in Nature Communications, but I still have some minor corrections to suggest:

We thank the referee for this very positive evaluation of the manuscript.

- I understand References 20-22 are supposed to be for exciton-polaritons in TMDC monolayers, as stated in the introduction where these references are called, but Ref. [20] is more about van der Waals heterostructures. But most importantly, there is an earlier paper on this topic missing: Nature Photonics 9, 30 (2015), which was published (on-line) about a year before Ref. [20]. It is worth to cite it along with 20-22

We thank the referee for spotting these two inconsistencies and have accounted for them in the revision, by including Nature Photonics 9, 30 (2015) as a reference and pointing the reader's attention to the fact, that Ref [20] demonstrates both strong coupling with a monolayer as well as a VdW heterostructure.

- It would be better to define the abbreviation DBR before its use.

We thank the referee, and corrected the manuscript in this regard.

- Equations are not numbered. The first equation in the manuscript is a matrix equation. Then, just after it, there is a determinant equation: is this equation correct? shouldn't it have "-E" added to the diagonal terms? Anyway, in may opinion, showing this step on the diagonalization of the matrix in first equation is unnecessary - it's standard procedure.

We agree with the referee and removed the determinant equation to circumvent redundancy. Equations are numbered in new manuscript.

Reviewer #3 (Remarks to the Author):

The authors demonstrated the strong light-matter coupling between the Tamm plasmons and the excitons in GaAs quantum wells and $MoSe_2$ monolayers which lead to the formation of hybrid polariton modes. The momentum resolved photoluminescence and reflectivity results clearly show the anticrossing feature of such hybrid system. This may be useful for the design of the electrical injection of hybrid polariton LED device or polariton laser. Before it is publishable, I have some minor comments about it shown below.

1. In Fig. 2b, is the calculated reflectivity spectrum stands for the empty cavity mode? The reflectance dip of it corresponds to 1.64 eV is different from the black dashed line in Fig 3a at k//=0 (about 1.633 eV), where is the redshift comes from?

The cavity mode was calculated by the original design parameters. However, due to naturally evolving uncertainties during the epitaxial growth, the experimentally extracted cavity mode (such as the fit in fig 3a) typically deviates somewhat from the ideal model. We included an explanation about this discrepancy in the revised manuscript.

2. minor mistake: the label of the last picture in Fig. 4 is “b” which should be “f”.

We thank the referee for spotting this.

3. In Fig 3 and Fig. 4, the authors compared two different temperatures 4 K/150 K and 4 K/140 K, it would be better if the high temperature is the same.

The data in fig 3 and fig 4 evolved from two different measurement runs in two different setups (optimized for angular resolution vs. spatial filtering). Unfortunately, as a result of technical difficulties (lack of liquid helium) the set of data which demonstrates the simple QW-polariton dispersion at 140 K is obsolete. However, since we provide the quantitative evolution of the QW resonance with temperature in the supplementary section of the manuscript, we hope that the Referee agrees with us that this solely cosmetic correction which would result in a further delay of publication is dispensable.

4. Page 6, the meaning of the coefficient should be explained.

We explain the meaning of the Hopfield coefficients in the revised manuscript.

5. Minor mistakes in Supplementary materials:
First paragraph: “(Fig. S2b)” should be “(Fig. S1b)”;
Third paragraph: “Fig. 2Sb” should be “Fig. S2b”.

We have fixed these mistakes.

Reviewer #4 (Remarks to the Author):

The manuscript is an experimental demonstration of hybridization between QW excitons and 2D excitons in MoSe2 mono-layers placed in the cavity. The work is highly relevant and well performed. I recommend publication provided the authors answer a few comments
(1) How does the fact that the excitons in MQW's have longer lifetime (narrow line-width) influence hybridization?

The longer lifetime of the MQW excitons result in a mismatch in the coupling strength, which we account for in the theoretical description of the hybrid polariton formation. However, we agree that it will be interesting to observe the dynamics in our system, since the III-V QWs will also provide a long living reservoir which is mostly absent in TMDC monolayers. We have put a not regarding this point in the conclusion of the revised manuscript.

(2) Will applying bias across the structure change hybridization as excitons go in and out of the resonance?

Biasing the structure will have two effects: First, it will detune the III-V excitons from the TMDC excitons, which can be used to tune the degree of hybridization. If tunneling sets in (above some voltage), the coupling strength of the III-V QWs will decrease which will also act on the hybridization.

The authors claim that the hybrid polaritons have advantages when it comes to the electrically pumped LED, but this advantage is not clear to me.

While direct electrical injection into monolayers is possible (ref 28 of the revised manuscript), we believe that the III-V platform still is preferable for integration into diode structures due to the very good electron and hole mobilities and well established processing technology for various decades.

A simple, indirect pumping TMDC excitons in a structure with a built-in LED (e.g. non-resonant or in the weak coupling) should also be feasible. However, the strong coupling conditions ensure a maximal re-absorption of the initially emitted light, which makes this kind of pumping particularily attractive. We have slightly modified the conclusion to make this point more clear.

Reviewers' Comments:

Reviewer #4:
Remarks to the Author:
The authors have addressed all my concerns as well other reviewers comments. The manuscript can now be published in Nature Comms.
